# Novel radiotherapy target definition using AI-driven predictions of glioblastoma recurrence from metabolic and diffusion MRI

**DOI:** 10.1038/s41746-025-01861-2

**Published:** 2025-08-07

**Authors:** Nate Tran, Tracy L. Luks, Yan Li, Angela Jakary, Jacob Ellison, Bo Liu, Oluwaseun Adegbite, Devika Nair, Pranav Kakhandiki, Annette M. Molinaro, Javier E. Villanueva-Meyer, Nicholas Butowski, Jennifer L. Clarke, Susan M. Chang, Steve E. Braunstein, Olivier Morin, Hui Lin, Janine M. Lupo

**Affiliations:** 1 Department of Radiology & Biomedical Imaging, University of California, San Francisco, CA USA; 2 UCSF/UC Berkeley Graduate Program in Bioengineering, University of California, San Francisco & Berkeley, CA USA; 3 Department of Neurological Surgery, University of California, San Francisco, CA USA; 4 Department of Radiation Oncology, University of California, San Francisco, CA USA

**Keywords:** Predictive markers, Translational research, Cancer imaging, CNS cancer

## Abstract

The current standard-of-care (SOC) practice for defining the clinical target volume (CTV) for radiation therapy (RT) in patients with glioblastoma still employs an isotropic 1–2 cm expansion of the T2-hyperintensity lesion, without considering the heterogeneous infiltrative nature of these tumors. This study aims to improve RT CTV definition in patients with glioblastoma by incorporating biologically relevant metabolic and physiologic imaging acquired before RT along with a deep learning model that can predict regions of subsequent tumor progression by either the presence of contrast-enhancement or T2-hyperintensity. The results were compared against two standard CTV definitions. Our multi-parametric deep learning model significantly outperformed the uniform 2 cm expansion of the T2-lesion CTV in terms of specificity (0.89 ± 0.05 vs 0.79 ± 0.11; *p* = 0.004), while also achieving comparable sensitivity (0.92 ± 0.11 vs 0.95 ± 0.08; *p* = 0.10), sparing more normal brain. Model performance was significantly enhanced by incorporating lesion size-weighted loss functions during training and including metabolic images as inputs.

## Introduction

Current standard-of-care (SOC) treatment of highly infiltrative glioblastoma (GBM) continues to begin with maximal safe surgical resection, followed by external beam radiotherapy (RT; with a total dose of 60 Gy in 2 Gy fractions over a course of 6 weeks) in conjunction with temozolomide (TMZ) chemotherapy^1^. Despite decades of clinical trials incorporating novel systemic and targeted agents and radiation dosing schemes, the only change to SOC treatment of GBM is the inclusion of Tumor-Treating-Fields upon completion of RT, which has resulted in minimal improvements in outcome beyond the typical 12–15 month dismal prognosis^2,3^. This is in part due to the difficulty in both identifying and treating the full extent of these highly infiltrative tumors with RT, while also sparing critical brain tissue to preserve normal brain function^4^.

While recent advances in RT delivery can provide millimeter-scale precision and dose modulation, current RT treatment planning protocols are still based on a uniform 1–2 cm geometric expansion of the gross-tumor-volume defined on conventional post-contrast T1-weighted and T2-weighted FLAIR MRI, without considering the spatial heterogeneity and infiltrative nature of this disease. This has the unintended consequences of undertreating subclinical disease not yet visible on anatomical MRI, as well as unnecessarily irradiating normal brain tissue, adversely affecting clinical outcome and increasing toxicity. While most tumor progression happens locally within the 2 cm expansion of the hyperintense lesion from T2-weighted images, partially due to the consensus tendency to overtreat^5^, tumor progression occurs beyond the 2 cm expansion target volume for about 10-37% of patients^6–10^, with more recent studies reporting upwards of 25%^11^. Up to 60% of irradiated tissue in the high-dose field is normal-appearing brain^12^, causing neurotoxicity, which can negatively affect a patient’s cognitive function, quality-of-life, and overall survival (OS)^13,14^. The introduction of anti-angiogenic agents also alters the pattern of tumor recurrence, with non-enhancing tumor progression becoming more prevalent than previously observed, further complicates target planning. Thus, novel approaches for defining RT target volumes based on knowledge of individual tumor recurrence patterns have great potential to improve outcomes for patients with GBM.

Recent advances in diffusion-weighted and metabolic MRI have enabled voxel-level visualization and characterization of cellular-level measures of tumor involvement^15,16^, yet are largely unused in RT planning outside of a few recent single-arm phase II clinical trials^17–22^. Increases in apparent diffusion coefficient (ADC) and decreases in fractional anisotropy (FA) using diffusion tensor imaging (DTI)^23–25^ can reflect subclinical tumor invasion, which causes an increase of edema and decrease in directionality along white matter tracts^26^. Metabolite levels estimated using proton Magnetic Resonance Spectroscopic Imaging (^1^H-MRSI) and the derived Choline-to-NAA index (CNI) can probe underlying cellular metabolism associated with infiltrative tumor^26,27^, hypoxia^28^, tumor progression, and survival^15,29,30^. Although these MRSI^17–19,22,31–33^ and other imaging methods such as ^18^F-FET-PET and ^11^C-MET-PET^34–37^ have shown great promise in more accurately predicting tumor infiltration for incorporation into RT planning, these studies have been limited to simulations or retrospective analyses, lacking prospective evaluation in a clinical trial. More recent prospective single-arm phase II studies have used imaging to either guide dose escalation based on ^18^F-DOPA-PET^38^ or choline/NAA > 2 from MRSI^18^, or boost regions based on elevated relative cerebral blood volume (rCBV; an MRI-based measure of the volume of blood in a given amount of tissue relative to that in normal-appearing white matter that can be used to reflect neovascularization in the tumor) or hypercellularity volume defined on high b-value diffusion images^39,40^. Although these studies demonstrated significant improvements in outcome (92% 12-month OS rate) compared to historical controls^39^, they relied on images of the tumor location prior to radiation, without modeling where the tumor would ultimately progress.

The goal of this study was to develop a novel, personalized approach for defining RT clinical target volumes (CTVs) utilizing machine- and deep learning-driven predictions of GBM recurrence patterns that incorporate pre-therapy diffusion-weighted and metabolic MR images. The resulting predicted volumes are then compared to the SOC 1–2 cm uniform expansion of anatomical lesion CTVs for their ability to cover the extent of the lesion at the time of recurrence. We hypothesize that this comprehensive strategy will result in a more biologically-relevant definition of RT target volumes based on the true extent of infiltrating tumor, which will more closely overlap with the region of progression, while minimizing the dose to normal brain.

## Results

### Patient characteristics

Median PFS and OS were 7.0 and 17.6 months, respectively, for the entire cohort (Table 1). Although a significant difference (*p* = 0.0012) in OS was observed between the SOC+bevacizumab (ATT) cohort (median = 20.3months) and SOC cohort (14.6), no significant difference was found in OS between the SOC+enzastaurin (ENZA) and either SOC (*p* = 0.22) or ATT (*p* = 0.14). There was significant difference in PFS among cohorts (*p* = 0.021). Table 1 also lists the number of patients who progressed via CEL progression, T2L progression, or both for each cohort individually and all patients combined. The percentage of patients who recurred within the T2L was 7% (1-ATT/1-ENZA/5-SOC), between the T2L boundary and 2 cm margin was 65% (19-ATT/20-ENZA/26-SOC), and beyond the 2 cm expansion of the original T2L was 29% (7-ATT/9-ENZA/13-SOC).

**Table 1:** Patient characteristics

Patient cohort	All patients	SOC	SOC+bevacizumab (ATT)	SOC+enzastaurin (ENZA)
**N**	101	44	27	30
**% Male**	66% (67/101)	59% (26/44)	67% (18/27)	77% (23/30)
Tumor volume* (cm^**3**^**)**				
Average/stdev	34.4 $\pm$ 30.5	34.5 $\pm$ 25.3	38.7 $\pm$ 44.5	30.0 $\pm$ 23.7
Median	26.4	26.6	21.0	27.4
Range	[1.2 – 153.6]	[1.2 – 106.6]	[2.0 – 153.6]	[4.29 – 82.5]
**EOR**				
GTR	35	20	6	9
STR	56	22	17	17
Biopsy	10	2	4	4
**Progression type**				
CEL progression	7	5	1	1
T2L progression	50	14	18	18
Both	44	25	8	11
**Age (year)**				
Median	53	53	54	57
Range	[25–77]	[27–77]	[28–75]	[25–70]
**OS (months)**				
Average/stdev	23.4 $\pm$ 24.3	18.0 $\pm$ 11.6	40.1 $\pm$ 43.6	19.7 $\pm$ 10.3
Median	17.6	14.6	20.3	17.9
Range	[5.8 – 139.7]	[5.9 – 63.9]	[5.8 – 139.7]	[8.7 – 54.0]
**PFS (months)**				
Average/stdev	8.2 $\pm$ 5.7	5.2 $\pm$ 3.3	13.0 $\pm$ 5.9	8.4 $\pm$ 6.1
Median	7.0	4.7	12.0	7.1
Range	[0.8 – 25.9]	[0.8–11.7]	[1.9 – 25.9]	[1.8 – 23.0]

^*^(T2L + CEL) at baseline scan.

*EOR* extent of resection, *GTR* gross total resection, *STR* subtotal resection, *CEL* contrast enhancing lesion, *T2L* T2 lesion, *OS* overall survival, *PFS* progression free survival.

### Statistical analysis

Statistically significant elevations in median nFLAIR, nADC, nFA, CNI, and CCr were observed between both NAV→CEL (*p* = 9.3 × 10^–14^, 7.3 × 10^–7^, 2.7 × 10^–6^, 1.1 × 10^–14^, 1.7 × 10^–13^, respectively) and NEL→CEL (*p* = 7.9 × 10^–21^, 1.6 × 10^–9^, 8.7 × 10^–8^, 5.5 × 10^–17^, 2.6 × 10^–16^, respectively) voxels compared to stable NAV voxels for all patients. Highly significant increases in median nFLAIR, CNI, and CCrI were observed for NAV→NEL (*p* = 1.5 × 10^–16^, 2.4 × 10^–15^, 2.8 × 10^–16^, respectively) voxels compared to stable NAV voxels, while increases in median nADC also reached a statistical significance of *p* = 0.0009. Supplementary Fig. 1 shows differences in imaging metrics between the various progressed and stable regions of all voxels within a 4 cm boundary of the original lesion for all the patients combined and by therapy group. The metabolic and diffusion parameters consistently predicted of infiltrative tumor across all cohorts.

### Machine learning voxel-wise predictions

**Fig. 1:**
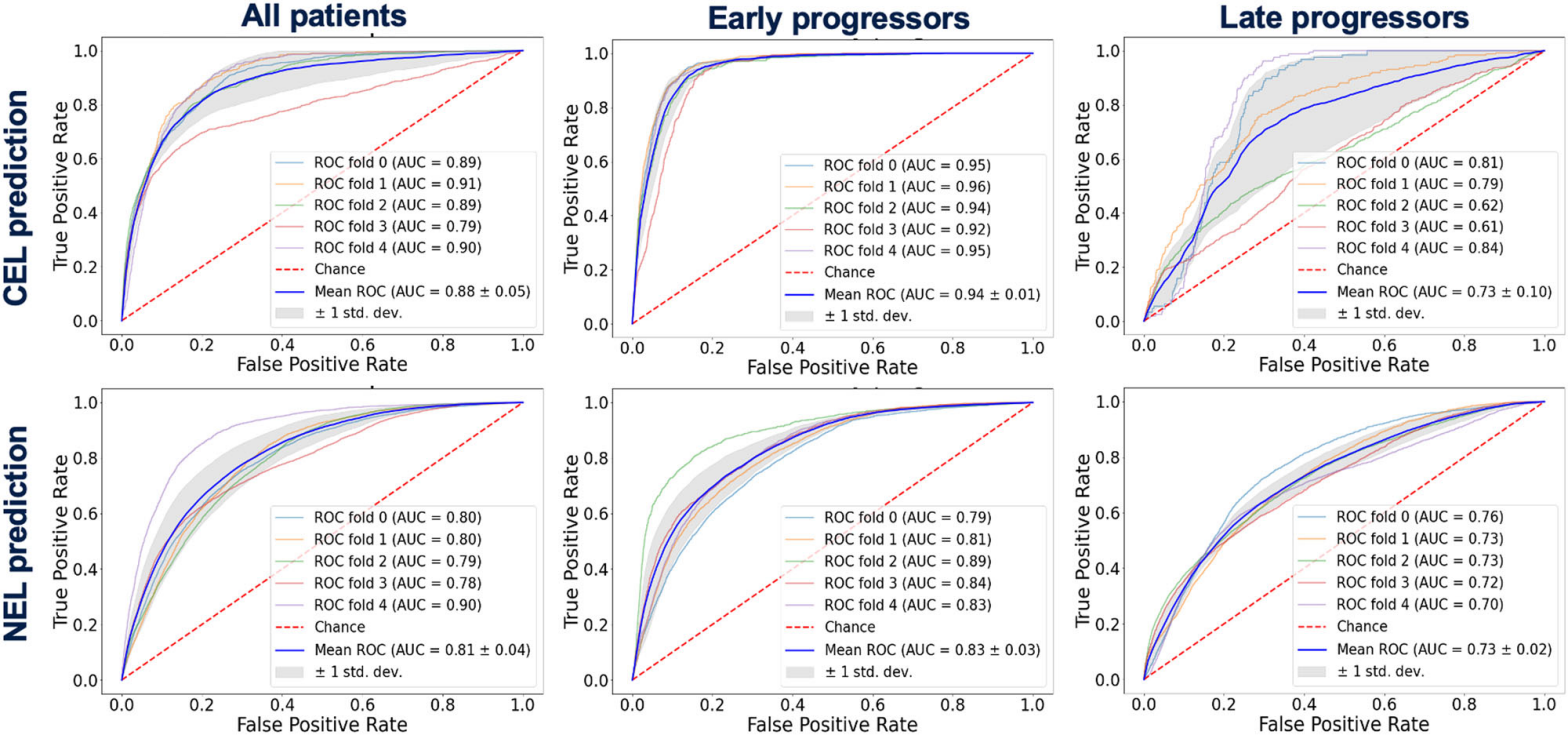
Results of fivefold cross-validation voxel-wise prediction models. Random forest (RF) models were able to predict contrast-enhancing lesion (CEL) progression (top row) and non-enhancing lesion (NEL) progression (bottom row). Highest area under the curve (AUC) was achieved for predicting early progressors (t < 7 months), especially for CEL recurrence. ROC=Receiver Operator Characteristic.

Figure 1 shows the five-fold CV ROC curves for voxel-based predictions of CEL (*top row*) and NEL (*bottom row*) at the time of progression. The best RF model that predicted NAV→CEL and NEL→CEL progression from originally stable NAV voxels achieved a mean AUC of 0.88 when combining all patients. When splitting patients by median time to progression (t = 7 months), the CEL-progression model performed very well for patients who progressed before 7 months (*n* = 50; AUC = 0.94), but resulted in reduced performance (*n* = 51; AUC = 0.74) for patients who progressed later. Similarly, the best RF model that classified stable (NAV→NAV) vs. non-enhancing (NAV→NEL) progression achieved a mean AUC of 0.81 when combining all patients. But again, when splitting patients by median time to progression, the NEL-progression model performed better for patients who progressed before 7months (*n* = 50; AUC = 0.83) than for patients who progressed later (*n* = 51; AUC = 0.73), although the difference was not as drastic as the CEL progression model. The most important features for the CEL-progression model for all patients were CNI, CCrI, nLipid, and nFLAIR, while the most important features for the NEL-progression model were CCrI, CNI, and nFLAIR.

### Benefit of including a personalized loss function based on lesion size

Varying ${\rm{\alpha }}$ and ${\rm{\beta }}$ based on lesion size in the loss function accounted for the class imbalance that resulted in patients with smaller lesions by determining more-optimal thresholds for tolerance in the model, improving its performance. Supplementary Fig. 2 illustrates the change in model performance when varying $\alpha$ and $\beta$ values of the Tyversky loss based on lesion size, with the optimal $\alpha$ and $\beta$ values occurring near the point where $\alpha$ approaches 0.02 and $\beta$ approaches 0.98, whereby $\alpha$ approximates the average percentage of tumor voxels to the total brain voxels. At this point, the sensitivity rapidly increased while the specificity remained relatively high. A sharp drop off in specificity then occurred above $\beta$ = 0.98. As shown in the validation performance during training, using the combination loss function (PCC + BCE or PCC + 0.5BCE) achieved the best performance, where the model was able to converge quickly. These loss functions had significantly higher sensitivity in model performance on the test set, at the expense of a small drop in specificity. Although including BCE with PCC loss slightly improved overall performance by increasing specificity (Supplementary Fig. 3), this combined loss function often resulted in non-contiguous segmentations, so PCC loss was selected for training our final model.

### Incorporating diffusion-weighted and metabolic imaging

**Fig. 2:**
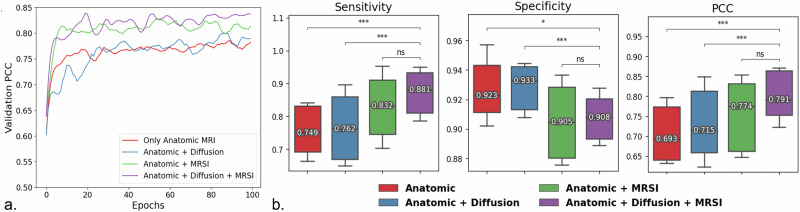
Model performance comparison among different MRI input modalities. **a** Validation Progression Coverage Coefficient (PCC) scores during training for all models; and **b** Sensitivity, specificity, and PCC metric comparison for patients in the test set. All models were trained using the PCC plus binary cross entropy (BCE) loss function. Wilcoxon signed rank tests were used with significant levels defined as *p* < 0.05, 0.01, and 0.001 and indicated by single, double, and triple asterisks, respectively. The model trained using all MRI modalities achieved a significantly higher PCC by improving the sensitivity of the model. MRSI Magnetic Resonance Spectroscopic Imaging.

A comparison of models trained using different combination of MRI modalities is displayed in Fig. 2. The model using anatomical+diffusion+MRSI achieved the most sufficient learning, as demonstrated by the validation curves in Fig. 2a. This was further confirmed during inference, where this model also achieved significantly higher sensitivity and PCC scores than models trained without MRSI metrics (Fig. 2b, left and right), while maintaining relatively high specificity (0.91), despite it significantly dropping from 0.93 (*p* = 0.03; Fig. 2b, middle). Models trained without MRSI (using only anatomic images and anatomic+diffusion images) did not achieve as optimal of performance during training and resulted in significantly lower sensitivity during testing (8.4–15.0%; *p* = 0.00024 and *p* = 0.00061 respectively), as well as exhibiting higher variability among patients (Fig. 2b, left). Test performance, as measured by PCC - which considers sensitivity, specificity, and lesion size - dropped by 9.6% and 12.4% when removing MRSI and both MRSI and diffusion metrics, respectively (*p* = 0.00021 and *p* = 0.00053; Fig. 2b, right).

### Model comparison among different treatment plans

**Fig. 3:**
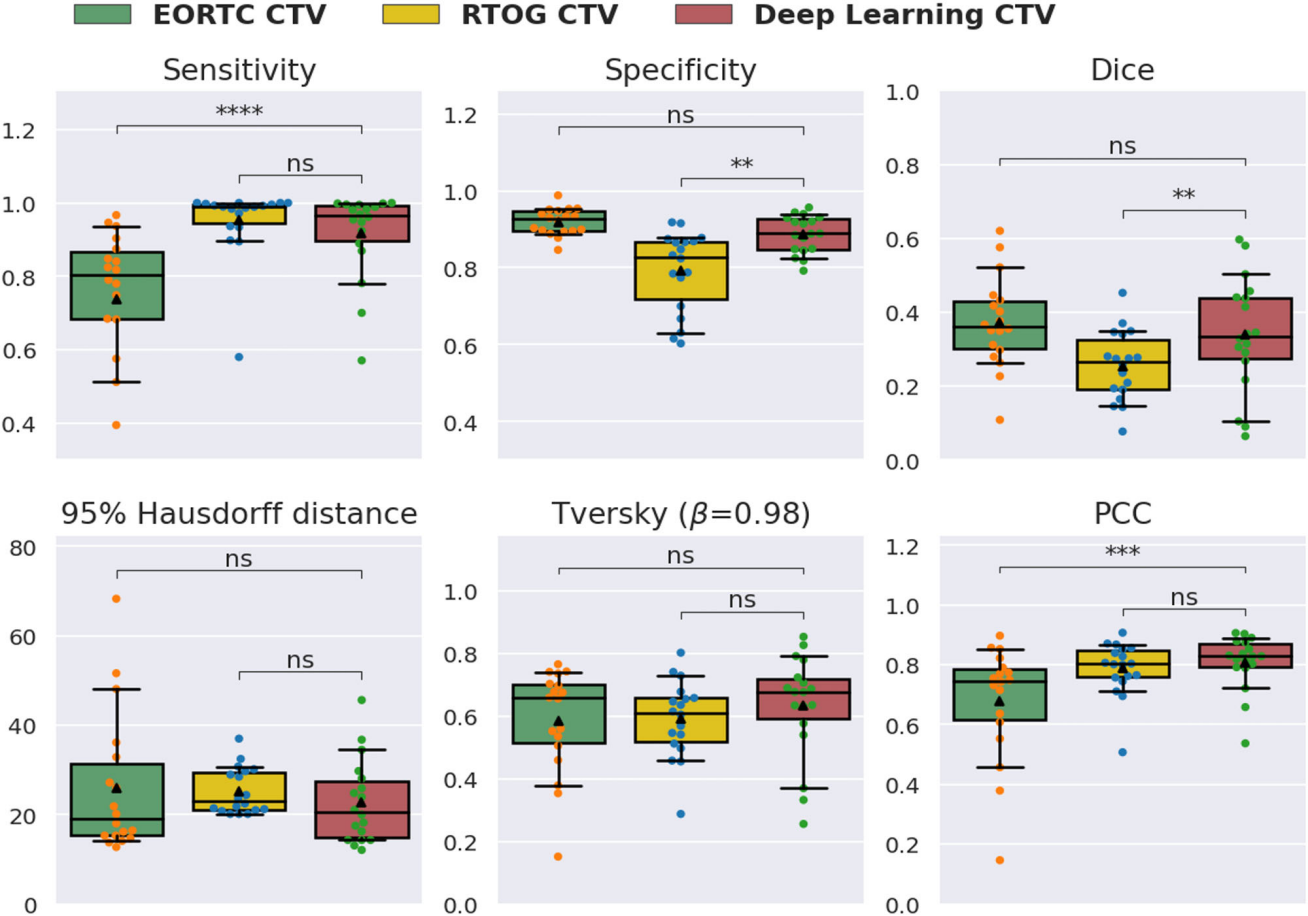
Model performance comparison among standard of care and deep learning generated target volumes. Our model showed a significantly higher median sensitivity and Progression Coverage Coefficient (PCC), and comparable specificity to the European Organization for Research and Treatment of Cancer (EORTC) clinical target volume (CTV). The deep learning model also resulted in a significantly higher median specificity and Dice score, and comparable sensitivity to the Radiation Therapy Oncology Group (RTOG) CTV. Wilcoxon signed rank tests were used with significant levels defined as *p* < 0.05, 0.01, and 0.001 and indicated by single, double, and triple asterisks, respectively.

Our best-performing model used anatomic+diffusion+MRSI as inputs, and was trained using a PCC loss function, with an initial learning rate of 5 × 10^–5^ using a Ranger optimizer and convergence at epoch 123. To further evaluate the predicted CTV generated using this model, we compared its performance against two hypothetical spatial CTVs according to the recommendations put forth by the EORTC^41,42^ and RTOG^43,44^. Figure 3 and Supplementary Table 1 summarize the average sensitivity, specificity, Dice score, 95% Hausdorff distance, Tversky coefficient, and PCC results of both hypothetical CTVs and our best deep learning predicted CTV for patients in the test set. Our best deep learning model trained using all 3 MRI modalities had comparable sensitivity (0.92 ± 0.11 vs. 0.95 ± 0.08, *p* = 0.09) and PCC (0.81 ± 0.09 vs. 0.79 ± 0.09, *p* = 0.09) to the more aggressive SOC RTOG CTV, and had a significantly higher specificity (0.89 ± 0.05 vs. 0.79 ± 0.11, *p* = 0.004; Fig. 3 and Supplementary Table 1). Compared to the more conservative EORTC CTV, our method had significantly higher sensitivity (0.92 ± 0.11 vs. 0.74 ± 0.21; *p* = 7.3 × 10^−6^), PCC (0.81 ± 0.09 vs. 0.68 ± 0.19; *p* = 0.0001), and similar specificity (0.89 ± 0.05 vs. 0.92 ± 0.03; *p* = 0.053). Supplementary Table 2 lists the individual patient performance for each CTV. Our deep learning approach exhibited improved performance in covering the region of progression compared to RTOG and EORTC CTV definitions, in 72% and 94% of the patients in the test set, respectively, while only missing more than 13% of the lesion in 3 patients. The addition of the anti-angiogenic cohorts did not reduce the overall performance in SOC patients, who exhibited a non-significant trend toward higher specificity and lower sensitivity, compared to patients who also received an anti-angiogenic agent, resulting in similar PCC values among cohorts (Supplementary Fig. 4).

**Fig. 4:**
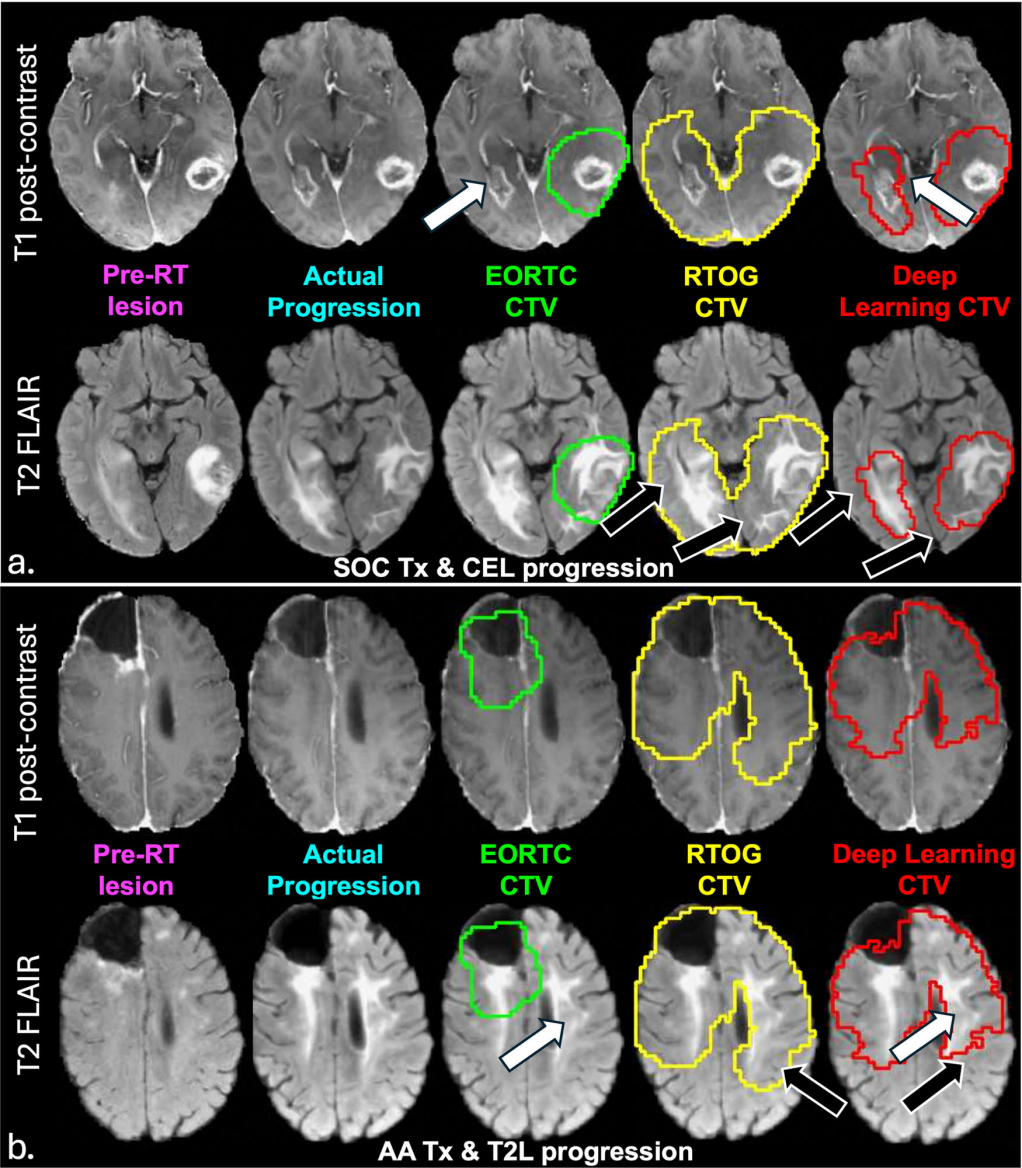
Comparison of CTVs for 2 example patients in the test set. **a** Example of contrast-enhancing lesion progression after a patient receiving standard-of-care (SOC) therapy (patient #16 of Supplementary Table 2). **b** Example of non-enhancing T2 lesion progression in a patient who received anti-angiogenic (AA) therapy in the form bevacizumab plus SOC (patient #2 of Supplementary Table 2). In both these patients, increased sensitivity to covering the progressed lesion (white arrows) was observed with our deep learning model (red contour) compared to the European Organization for Research and Treatment of Cancer (EORTC) defined clinical target volume (CTV) (green contour), while increased specificity was achieved compared to the Radiation Therapy Oncology Group (RTOG) defined CTV (yellow contour), demonstrating the ability of our approach to spare more unaffected brain (black arrows).

Visual comparison of deep learning and clinical CTVs for 5 example patients in the hold out test set are shown in Fig. 4 and Supplementary Fig. 5, with panels a and b of Fig. 4 corresponding to patients #16, and 2 in Supplementary Table 2, and panels A, B, and C of Supplementary Fig. 5 corresponding to patients #1, 15, and 13 in Supplementary Table 2. The patients in Fig. 4 were selected to highlight model performance in terms of both improved sensitivity or coverage of progression (white arrows) and specificity or sparing of normal brain tissue (black arrows), for representative patients exhibiting contrast-enhancing and non-enhancing lesion progression after SOC and bevacizumab + SOC, respectively. Visually, the predicted deep learning CTV (red contour) covered the entire tumor region on the progression scan in both examples, covering the region of new contrast enhancement that was missed by the EORTC CTV (green contour) in Fig. 4a, while simultaneously not overtreating beyond the progressed T2-lesion and sparing more normal brain tissue compared to the RTOG CTV (yellow contour). In the patient in Fig. 4b who experienced progression based on a growing T2 lesion, our deep learning CTV was again able to cover the large portion of the T2 lesion missed by the EORTC CTV, while sparing more normal brain than the RTOG CTV. The patient shown in Supplementary Fig. 5a who progressed by increases in both contrast-enhancing and non-enhancing lesions, demonstrated our multimodal MRI deep learning generated CTV’s (red contour) ability to outperform both the EORTC (green contour) and RTOG (yellow contour) defined CTVs, with the highest sensitivity, specificity, and PCC. Although the sensitivity of all models was >0.95 for the patient in Supplementary Fig. 5b who progressed via contrast-enhanced lesion, our model achieved 100% sensitivity with comparable specificity to the EORTC CTV (0.88 vs. 0.90), significantly reducing the extent of high-dose radiation to normal brain tissue (black arrows). Even in the patient example with worst model performance (Supplementary Fig. 5c) in terms of both sensitivity and PCC, our deep learning CTV still demonstrated improved specificity, while having the same sensitivity to the EORTC and RTOG CTVs that similarly missed covering a large portion of the progressed lesion.

## Discussion

Although recent developments in the field of radiotherapy have allowed empirical doses to be delivered precisely to the planned CTV, SOC-RT treatment is still only planned using the tumor volume defined from anatomical T1-weighted and T2-weighted MRI, plus a uniform, isotropic 2 cm expansion of the gross tumor volume. This practice fails to acknowledge the spatial heterogeneity and anisotropic directionality of tumor infiltration into normal brain tissue. While tumor progression often occurs locally, previous studies have shown that tumor can spread beyond the SOC CTV in approximately 10–37% of patients^6–10^, which is consistent with our dataset, where 28% of all patients had tumor progression outside of the more spatially aggressive SOC ROTG CTV. This indicates that anatomically-normal disease is still undertreated in the current paradigm for RT planning. At the same time, the ROTG CTV has low specificity, with approximately 60% of high dose voxels being normal brain tissue^12^, which can lead to cognitive decline, reduced quality of life, and in extreme cases, shortened overall survival^13^. Although recent advances in diffusion-weighted MRI and metabolic ^1^H-MRSI^15^ have allowed the visualization and detection of subclinical tumor cells in patients with GBM, they remain underutilized in clinical RT planning, apart from a few research studies^17–22^. We have previously shown^30^ that voxel-wise abnormalities in ADC, FA, and CNI can be combined to predict future tumor progression, with higher ADC, lower FA, and higher CNI all denoting regions at higher risk of progression. This study expands on that knowledge by: (i) incorporating deep learning to guide precision-based RT planning, (ii) utilizing cutting edge technology for more accurate inter-exam image registration, specifically trained on longitudinal glioma scans to account for tissue shift, (iii) developing new, more appropriate metrics for quantifying loss in training and evaluating results to improve prediction accuracy; (iv) demonstrating clinical utility in three unique upfront chemotherapy treatment regimens; and (v) comparing performance to the two most utilized clinical standards for CTV definition.

Our statistical analyses of DWI and MRSI metrics have confirmed that pre-RT DWI and MRSI can identify subclinical disease that appears normal on anatomical MRI, especially on CNI and CCrI maps. Significant differences in the median normalized T2-FLAIR signal between normal voxels that remained stable and progressed suggest that subtle differences in normalized T2-FLAIR hyperintense signal (that appear normal visually) may also indicate abnormal tissue. Diffusion-weighted metrics also proved valuable in distinguishing between stable normal-appearing voxels and voxels that progressed to become contrast-enhancing (from both originally normal-appearing and non-enhancing voxels), but were less effective in predicting non-enhancing T2-FLAIR progression. This is likely due to these voxels having similar diffusion values to normal-appearing brain. These results aligned with previous findings described by Anwar et al.^30^ in a much smaller cohort and were consistent across all therapy groups.

Our RF model further supported our hypothesis that advanced MRI can help predict regions of future anatomically-defined progression, achieving an average ROC-AUC of 0.88 for the CEL-progression model and 0.81 for NEL-progression. The superior performance in CEL-progression prediction was expected, given the similar intensity of those voxels to the pre-RT CEL with more abnormal MRSI and diffusion-weighted features. Lipid was a significant predictor only for predicting CEL progression, likely due to elevated cell membrane breakdown resulting in detectable mobile lipids in areas experiencing blood-brain-barrier disruption preceding the presence of contrast enhancement. Lactate, however, was not a significant predictor of either CEL or NEL progression, suggesting that hypoxia may be a consequence rather than a precursor of tumor spread. Although diffusion-weighted imaging metrics were shown to be significant predictors of both NEL and CEL progression, neither ADC nor FA were among the most important features for the RF. This is likely due to heterogeneity in tumor microenvironments and partial volume effects, where neighboring voxels could have elevated and reduced nADC compared to normal, highlighting a potential pitfall of voxel-wise analyses. In patients that progressed earlier, it was easier to predict the location of voxels that recurred by both contrast-enhancing and non-enhancing progression models, consistent with previous publications that were able to use MRI markers to predict early progression but struggled to predict progression that occurred after 6 months^20^. When anti-angiogenic drugs (especially bevacizumab) were part of the treatment regimen, which was the case for 65% of patients, the classic MRI marker for progression (contrast enhancement) was obscured, resulting in delayed, often non-enhancing progression that inflated the false positive rate of the RF model.

Our deep learning-based CTV outperformed both SOC CTVs in covering the progressed lesion, demonstrating significantly higher sensitivity than the EORTC CTV and significantly higher specificity than the RTOG CTV, while maintaining comparable performance in other metrics. The RTOG CTV achieved the highest sensitivity (0.95 ± 0.08) due to the inclusion of the entire T2-FLAIR lesion plus an additional 2 cm margin, but also suffered from having the lowest specificity (0.79 ± 0.10), indicating that a high dose of radiation was delivered to many areas where tumor progression did not occur, overtreating normal brain tissue. On the other hand, the less aggressive EORTC CTV achieved relatively low average sensitivity for our patient cohort (0.74 ± 0.21), and was highly variable among patients. Our deep learning-based CTV outperformed both SOC CTVs in covering the progressed lesion, with significantly improved sensitivity (0.92 ± 0.11 vs. 0.74 ± 0.21, *p* = 7.3 × 10^–6^) but similar specificity (0.89 ± 0.05 vs. 0.92 ± 0.03, *p* = 0.053) to the EORTC CTV, and significantly higher specificity (0.89 ± 0.05 vs. 0.79 ± 0.10; *p* = 0.004) with comparable sensitivity (0.92 ± 0.11 vs. 0.95 ± 0.08, *p* = 0.09) to the more aggressive RTOG CTV. Our deep learning CTV also had a significantly higher PCC than that of the EORTC CTV, similar to that of the RTOG CTV, while also showing less variation among patients, as indicated by smaller variances observed for nearly all performance metrics with the deep learning CTV. Unlike the voxel-based predictions in Fig. 2 where model performance was correlated with time to progression, there was not a significant difference in overall model performance (based on PCC) in predicting progressed voxels between early (PCC = 0.803) and late (PCC = 0.815) progressors in our test set with our deep learning model. This further supports the benefit of our approach over voxel-based methods.

A key challenge in this study were designing optimal loss functions for training and metrics for evaluating a segmentation-based lesion prediction task. In a typical lesion segmentation task, where the main goal is to delineate the borders of the tumor based on boundaries defined at the current time point at which imaging is obtained, both false positives and false negatives can be penalized equally, making the Dice coefficient a suitable choice as both the loss function and evaluation metric. In our unique strategy of incorporating a segmentation task for voxel-based predictions over time, however, a low sensitivity would lead to undertreating the tumor, while low specificity would result in over-irradiating normal brain tissue. As a result, loss function modifications to account for tumor size were necessary to better balance sensitivity and specificity in a way that was clinically relevant. By varying the $\beta$ values of the Tversky loss (Supplementary Fig. 2), we found that the optimal Tversky’s $\alpha$ and $\beta$ occurs near where $\alpha$ approaches 0.02, the average percentage of tumor voxels within the brain mask. Dynamically altering $\alpha$ and $\beta$ based on the individual patient’s tumor size when calculating PCC loss also played an important role in correcting the intrinsic class imbalance, as patients with smaller lesions had a higher class-imbalance, requiring a higher $\beta$ to further reduce false negatives and improve sensitivity (Supplementary Fig. 3). Tumor location can also potentially influence tuning $\beta$ if tumors in certain locations are associated with local or distance recurrence. In glioblastoma, there is some evidence of location-based recurrence, whereby tumors that border with the subventricular zone are more likely to result in distance recurrences^45^ and therefore would not require as high of a beta value to mitigate the class imbalance problem inherent between the relatively smaller number of lesion voxels compared to the rest of the brain. Although we imagine that this effect would be quite minimal compared to the effect of variations in lesion size driving this imbalance, future studies that extend our approach to high grade IDH-mutant tumors that tend to be more frontal in origin will evaluate the influence of tumor location on $\beta$.

The importance of evaluation metric selection is underscored by the fact that the CTV that was modeled using only the pre-RT anatomical lesions had the highest Dice score as well as highest positive predicted value, mostly due to having low number of false positive voxels, yet was considered the poorest model by any measure as it neglects to cover any infiltrating tumor cells that later progress. This is reflected by the model’s extremely low sensitivity. The Tversky and PCC scores for this CTV were both low, reflecting its poor performance (Supplementary Table 1). In contrast, both SOC CTVs had low Dice scores, but comparatively higher Tversky ($\alpha$=0.02) and PCC scores. Our PCC metric additionally allowed for a personalized weighting when balancing the tradeoff between sensitivity and specificity during both training and evaluation, whereby patients with large tumors benefitted from weighting towards higher specificity (to prevent against overtreating normal brain), while patients with small tumors benefitted from enforcing a higher sensitivity (to ensure complete coverage of the progressed lesion). This personalized, yet quantitative approach for CTV definition is another advantage of this method over others that have tried to either create CTVs based on universal thresholds of imaging markers or subjectively create the CTV manually^17,39,40,46^.

When training and optimizing models using the same loss function, the multimodal anatomical+diffusion+MRSI model achieved significantly better performance compared to both the anatomical model and the anatomical+diffusion model, but only slightly better than the anatomical+MRSI (Fig. 2). This phenomenon can be partially explained by the training curves in Fig. 2a, whereby models that included MRSI metrics quickly converge in all instances, demonstrating their strong predictability power. Models trained with just anatomical+MRSI metrics, however, become less stable with training time, mostly because MRSI often did not cover the entire brain, leading to inaccurate predictions outside of the PRESS box where MRSI was acquired. The addition of diffusion images at this point helped in stabilizing performance at later training epochs. Although coverage of MRSI was a limitation at the time of data collection in this retrospective study, more recent improvements in MRSI acquisition methods including the implementation of automated, optimized prescriptions and SAT band placements, along with undersampling schemes and parallel imaging reconstructions, have resulted in whole- or nearly whole-brain coverage of brain MRSI in clinically feasible scan times of less than 12 min^28^. Using MRSI metrics as inputs to our model is both a strength and limitation of this study because: (1) it is not routinely performed in clinical practice, which could limit the widespread adoption of our model in the future, and (2) it has lower resolution than the other images used. However, since the acquisition of this data, higher resolution MRSI has become more widely available within clinically reasonable scan times, and open-source packages are available for post-processing^28,47–49^. Access to AI-based models and standardized commercial software for image registration, tumor segmentation, and metabolic imaging analysis are becoming increasingly important to readily incorporate these advances outside of a research setting. More recent advances in diffusion-weighted imaging such as restriction spectrum imaging along with multi-compartmental modeling of tissue microstructure, should similarly also be evaluated as a potential replacement for MRSI in future studies as they are easier to acquire in clinical settings.

Despite the promising results, several other limitations should be acknowledged. While our dataset is much larger than most studies, it is still considered small for deep learning tasks. Since our retrospective dataset included patients who were diagnosed before common prognostic molecular markers such as IDH mutational or MGMT-promotor methylation status, were routinely performed on most patients we could not consider their implications on model training or evaluation. Although combining patients from 3 different treatment cohorts, could be considered a weakness if anti-angiogenic agents impacted the performance, we found the network was able to learn important features of progression by both contrast-enhancing and T2-hyperintensity, potentially improving its generalizability to other patients as new therapies arise. The most important imaging features predictive of progression were also the same for all cohorts and the addition of patients treated with antiangiogenic drugs in training improved the test results for the SOC cohort. Furthermore, no significant decreases in performance were observed in the SOC cohort compared to the anti-angiogenic therapy cohorts across all evaluation metrics when using a model trained on all patients, demonstrating the benefit of a larger dataset outweighing the difference in patient cohort for this deep learning task. Potential errors in correcting tissue shift during image alignment may have affected the accuracy of voxel-wise analyses, although a novel state-of-the-art co-registration algorithm^49^ designed specifically for this purpose was employed and visual quality checks were conducted for each case. Furthermore, additional non-rigid registration was implemented when necessary and all images were downsampled to a 3mm^3^ voxel size to further minimize the impact of any residual errors in alignment. While the sensitivity and specificity of our model were higher than any models from other similar studies, and higher compared to both standard-of-care CTVs, we have yet to determine how this superior performance, in terms of both sparing of normal brain tissue and treating infiltrative disease, will translate into improved outcomes (survival and quality-of-life) when applied clinically. Although we have demonstrated the potential of utilizing Tversky-based coefficients and PCCs as loss functions and evaluation metrics for designing personalized target volumes, their ultimate clinical utility also needs to be validated in a larger, external cohort. Despite our inability to incorporate dose maps into this analysis because many of our patients were treated at different institutions where this information was not available, the goal of this work was to provide a region where radiation should be delivered, not the dose distribution. As both higher specificity within the T2L + 2 cm and higher sensitivity beyond the T2L + 2 cm margin were found with our deep learning-based approach, it can potentially help improve future target definition for guiding where the higher 60 Gy dose can be delivered, while simultaneously treating areas not in our predicted volumes but within the T2L + 2 cm margin with a lower dose, personalizing RT.

In conclusion, our study demonstrated the ability to use pre-treatment diffusion-weighted imaging and ^1^H-MRSI, along with machine learning and deep learning techniques, to predict future regions of tumor progression and automatically generate clinical target volumes for RT planning. For the deep learning segmentation task, we found that the best model was trained using a novel loss function that improved model performance by taking the size of the original tumor into consideration, and that incorporated diffusion-weighted imaging plus ^1^H-MRSI. Our deep learning derived CTV using multi-parametric MRI was better at covering regions of subsequent progression than CTVs generated in standard clinical practice using the recommendations of the EORTC and the RTOG, suggesting that our approach has the potential to assist future personalized RT planning. Future studies will prospectively validate these findings in additional cohorts that include patients who were originally treated according to the most recent EORTC and RTOG guidelines before incorporating this approach in a clinical trial.

## Methods

### Patient characteristics and study design

A total of 101 patients, who were newly-diagnosed from 2002 to 2012 with a pathologically confirmed primary GBM according to WHO 2016 criteria and enrolled in a longitudinal, research MR imaging study, were included in this retrospective analysis. All patients received SOC treatment, including surgical resection followed by external beam RT (total dose of 60 Gy in 2 Gy fractions over a course of 6 weeks), concomitant daily TMZ (75 mg/m^2^), and six cycles of maintenance adjuvant TMZ chemotherapy (total 150–200 mg/m^2^). Of these 101 patients, 44 received no additional treatment, while the rest were treated with an additional anti-angiogenic agent, either: (1) enzastaurin (250 mg daily, beginning at the start of RT; *n* = 30) or (2) bevacizumab (10 mg/kg every 14 days starting in week 2 of RT) plus erlotinib (150 mg/day continuously or 500 mg/day continuously if on anti-epileptic drugs starting on day 1 of RT, *n* = 27), as summarized in Table 1. All patients gave informed consent to participate in research according to guidelines established by the Institutional Review Board (IRB) of the University of California, San Francisco (UCSF), in accordance with the Declaration of Helsinki. The study was approved by UCSF’s Helen Diller Family Comprehensive Cancer Center’s (HDFCCC) CNS site committee, the HDFCCC’s Protocol Monitoring Review Committee, and UCSF’s IRB (protocol #22-37795).

All patients received a baseline MRI scan (post-surgical resection but within 1 week prior to initiating radiotherapy and chemotherapy) that included T2-FLAIR imaging, pre- and post-contrast T1-weighted imaging, DWI, and MRSI. After the course of radiotherapy and chemotherapy, patients were followed serially with clinical MRI scans every two months (including at least pre- and post-contrast T1-weighted and T2-FLAIR imaging) until progression. Given the retrospective nature of this study, pseudoprogression, defined as an increase in tumor size observed on imaging that is due to the effects of treatment rather than actual tumor growth, was not an issue because: (1) 50% of the patients who received SOC therapy had pathologically confirmed progression, while the other half were confirmed by sustained increases on follow-up imaging according to RANO criteria^50^; and (2) for the other 2 cohorts with anti-angiogenic drugs on board, the concern becomes pseudoresponse potentially delaying the time of progression in these cohorts compared to the SOC, even though overall survival was similar in the overall trials.

### Image acquisition

Pre-RT MR examinations were performed on a 3 T GE Signa scanner (GE Healthcare, Waukesha, WI, USA) using an eight-channel phased-array head coil. Standard anatomical imaging included T2-weighted FLAIR and 3D T1-weighted IR-SPGR imaging pre- and post- the injection of a gadolinium-based contrast agent. For pre-RT MRI scan, diffusion-tensor images (DTI) were obtained with b = 1000 s/mm^2^, 6-directional diffusion-weighted echo-planar imaging sequence, and 4 b_0_ images (repetition-time[TR]/echo-time[TE] = 1000/108 ms, voxel size=1.7–2.0 × 1.7–2.0 × 2.0–3.0 mm). Lactate-edited 3D ^1^H-MRSI was acquired using point-resolved spectroscopy (PRESS) volume localization and very selective saturation (VSS) bands to avoid chemical shift artifacts as well as to suppress residual lipid signals (excited volume = 80 × 80 × 40 mm, TE/TR = 144/1100-1250 ms, overPRESS-factor = 1.5, nominal voxel size = 1 × 1 × 1 cm, flyback echo-planar readout in SI, total acquisition time = 9.5 min, sweep-width=988 Hz, and 712 dwell-points).

### Image and spectral processing

Anatomical regions of interest (ROIs) included the T1 contrast-enhancing lesion (CEL), T2 FLAIR hyperintensity lesion (T2L), non-enhancing lesion (NEL; defined as CEL subtracted from the T2L) and normal appearing voxels (NAV; defined as normal brain tissue from a skull-stripped brain mask obtained using the HD-BET brain extraction tool^51^ after subtraction of cavity, ventricles, and lesion ROIs). CEL, T2L and NEL ROIs were semi-automatically delineated on the pre- and post- contrast T1-weighted images (CEL) and T2-weighted FLAIR images (T2L), using in house software, before manual inspection and editing by a trained senior research specialist in radiology (TLL). All exams in the test set, as well as whenever there was a question on the boundary, were also verified by study neuroradiologist (JEVM). From the DTI data, maps of ADC and FA were calculated using FMRIB’s Diffusion Toolkit^52^ and normalized to the mode of intensities in normal-appearing brain tissue. Spectroscopic data were reconstructed and postprocessed using in-house software to generate metabolite peak heights maps, choline-to-NAA index (CNI), choline-to-creatine index (CCrI), and creatine-to-NAA index (CrNI) from baseline-subtracted, frequency- and phase-corrected spectra on a voxel-by-voxel basis^28,47^.

### Image alignment

All images from the pre-RT timepoint were rigidly aligned to the post-contrast T1-weighted image using Slicer’s BRAINSFit tool with B-spline warping^53^, or FMRIB’s FLIRT^54,55^, before being resampled to 3 × 3 × 3 mm resolution to mitigate any potential errors due to any residual misalignment. In order to allow for accurate matching of voxels between pre-RT and progression scans, a deep learning method specifically trained on serial post-resection glioma data with tissue shift^56^ as part of the BraTS-Reg 2022 challenge was utilized to align anatomical images at progression to those from the pre-RT scan, and the resulting transformation matrix was applied to all images and ROI files from the progression scan. This software, that ranked 1st place in the 2022 MICCAI BraTS-Reg challenge, utilized a 3-step deep-learning based approach to match voxels between pre-treatment and progression scans that consists of: (1) multi-level affine pre-alignment, (2) a conditional deep Laplacian pyramid image registration network (cLapIRN) with forward-backward consistency constraints, and (3) a non-linear instance optimization with inverse consistency. The resulting transformation matrix was then applied to all images and ROI files from the progression scan. All outputs were visually inspected by a senior research scientist (TLL) with over 20 years’ experience in verifying serial alignments. In the few cases where the quality of alignment was deemed not sufficient (<5%), non-rigid registration with B-spline warping using Slicer’s BrainsFit^46^ and intermediate scans was first applied, followed by the deep learning model. We found that this process was able to adequately handle alignment and any tissue shift from the shrinkage of the cavity visually.

### Voxel-based categorizations, analyses, and predictions

To assess significant differences between stable normal voxels (NAV→NAV, normal voxels pre-RT that remained normal at the time of progression) and voxels that progressed (either NAV→NEL, normal voxels pre-RT that became NEL at progression; NAV→CEL, normal voxels pre-RT that became CEL at progression; or NEL→CEL, NEL voxels pre-RT that became CEL at progression), the median value of each parameter map for each voxel category was calculated per patient, ensuring a minimum of 5 voxels for each group. Only voxels within a 4 cm expansion of the pre-RT T2-lesions were evaluated to mitigate class imbalance since there was no progression beyond this distance. The reason for this was twofold: (1) this approach prevented against artificially high AUCs in our voxel level predictions due to including a disproportional number of normal brain voxels compared to the lesion; and (2) 4 cm was determined by our clinicians (SEB) to be the furthest distance from the lesion that they would feel comfortable treating based on the results of an AI-based model. A Mann–Whitney signed-rank test was applied to compare median parameters between normal and progressed voxel groups (NAV, CEL, NEL). Statistical significance was determined as a *p* value of <0.05, with *p* values reported as *p* < 0.05, 0.01, 0.001, or 0.0001.

**Fig. 5:**
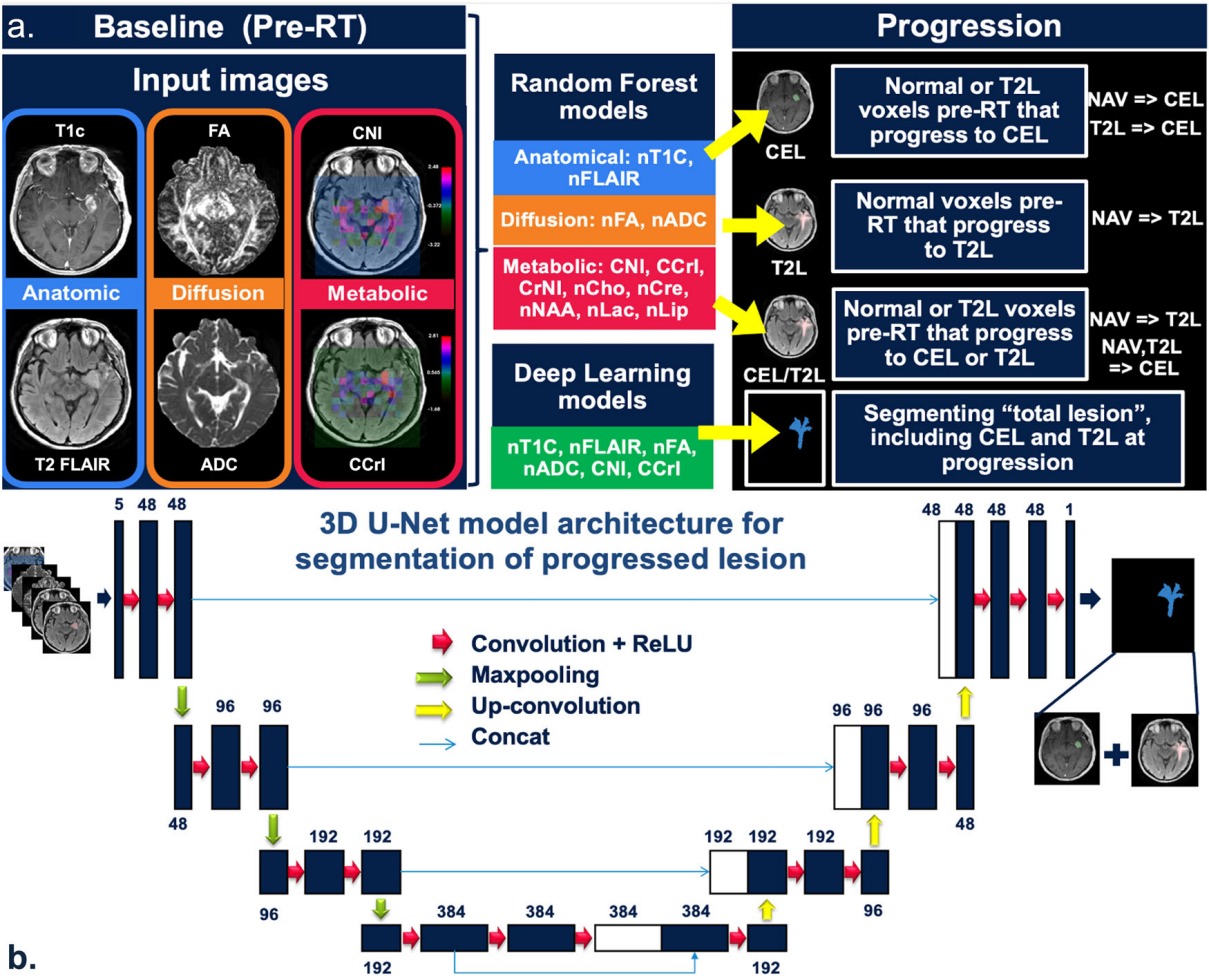
Overview of study approach and model training. **a** **Study schema**: Multi-parametric MR images from the baseline scan were used as input for both Random Forest (RF) and deep learning models. Three RF models were trained to predict the contrast-enhancing lesion (CEL), non-enhancing lesion (NEL), or CEL plus NEL progression. The deep learning model was trained to segment the entire lesion from both the pre-radiation therapy (RT) and progression time points combined in order to define the target volume for radiotherapy; **b** **Deep learning model architecture**: The chosen network used an encoder-decoder architecture, heavily inspired by the 3D U-Net architecture. The encoder had four stages, each consisting of two 3 × 3 × 3 convolutions. The decoder part of the network was almost symmetrical to the encoder. Shortcut connections between encoder and decoder of the same stage were performed by concatenation. T1c = T1-weighted image post-contrast; nT1C normalized T1c, FLAIR FLuid Attenuated Inversion Recovery, nFLAIR normalized FLAIR, NAV normal-appearing voxels, T2L = T2 lesion, FA Fractional Anisotropy, nFA normalized FA, ADC Apparent Diffusion Coefficient, nADC normalized ADC, CNI choline-to-NAA index, CCrI choline-to-creatine index, CrNI creatine-to-NAA index, nCho normalized choline, nCre normalized creatine, nNAA normalized NAA, nLac normalized lactate, nLip normalized lipid, ReLU Rectified Linear Unit, Concat concatenate.

To perform voxel-wise predictions of progression, we trained and tested two separate random forest (RF) models to distinguish: (1) NEL progression (NAV→NEL) and (2) CEL progression (NAV→CEL or NEL→CEL) from stable NAV (NAV→NAV). The inputs for each model included normalized anatomical images (nT1c, nT1, nT2-FLAIR); normalized DWI images (nADC, nFA), and normalized MRSI maps (nCho, nCre, nNAA, nLac, nLip, CNI, CCrI, CrNI). Each model was trained and tuned to maximize the area-under-the-curve (AUC) of the receiver operating characteristic (ROC) curve using five-fold cross-validation (CV) with a patient-wise stratified splitting method (train/test ratio of 70/30%). For each fold in the CV split, we ensured that voxels from the same patient could not co-exist in both training and test folds. To investigate the role of time-to-progression in our voxel-wise progression predictions, patients were stratified based on the median progression time (7 months) into early (progression < 7 months) and late (progression > 7 months) progression groups. The same RF model training approach was then repeated for each sub-group (Fig. 5a).

### Deep learning-driven approach for CTV generation based on recurrence predictions

A deep learning-based encoder-decoder model typically for in segmentation tasks was also trained to generate hypothetical target volumes because voxel-based RF models resulted in non-contiguous regions despite their high performance accuracy and CTVs require a contiguous closed shape. Since CTVs need to include both current tumor voxels and normal-appearing voxels based on anatomical imaging that are predicted to progress, we designed the deep learning task to predict a composite lesion mask of the NEL and CEL from both pre-RT and progression time points using only images from the pre-RT MRI scan. This task was performed using a 3D 4-staged U-Net architecture^57,58^ as depicted in Fig. 5b. Maps of nADC, nFA, CNI, CCrI, nT1C, and nT2FLAIR from the pre-RT time point were selected as model inputs, as they achieved the highest significance in distinguishing progressed voxels in both statistical analyses and machine learning predictions. All models used a 67/16/18 training/validation/test split of patients, with stratified splitting to ensure an equal proportion of treatment group in each split, and were trained using maps of nADC, nFA, CNI, CCrI, nT1C, and nT2FLAIR at the pre-RT time point as input images.

### Loss function modifications, optimization, and evaluation

To assess the impact of loss functions on model performance, we selected and evaluated four distinct loss functions: (1) standard Dice loss; (2) Tversky loss; (with varying $\alpha$ and $\beta$ values); (3) a novel individualized Progression Coverage Coefficient (PCC) derived from Tversky loss, but dynamically adjusting $\alpha$ and $\beta$ values for each patient depending on tumor size; and (4) a compound loss function combining the PCC loss with binary cross-entropy (BCE) loss. Our rationale for using PCC loss and metric stems from the observations that patients with smaller lesions present a more imbalanced dataset that requires a higher $\beta$ value to reduce false negatives and enhance sensitivity. By modulating $\alpha$ and $\beta$ based on lesion size, we aimed to this imbalance issue and determine more optimal decision thresholds for the model, potentially improving its performance. To specifically optimize $\beta$ during training, we incrementally adjusted $\beta$ in the $\beta$-dependent-Tversky loss while maintaining all other hyper-parameters constant. Our hypothesis was that the optimal Tversky loss function for balancing sensitivity and specificity would be achieved according to Eq. 2 by resolving the class imbalance problem.

1 $${{PCC}}=\frac{{TP}}{{TP}+\alpha {FP}+\beta {FN}}{\rm{where}}\,\beta =\frac{1}{f+1},\alpha =1-\beta ,{\rm{f}}=\frac{{n}_{{lesion}{{\_}}{voxels}}}{{n}_{{brain}{{\_}}{voxels}}}$$

2 $$\beta \sim 1-\frac{{n}_{{lesion}{{\_}}{voxels}}}{{n}_{{brain}{{\_}}{voxels}}\,+\,{n}_{{lesion}{{\_}}{voxels}}}$$

To assess the impact of each imaging modality on model performance, we trained and tested models using different combinations of image inputs using the optimized PCC + BCE loss: (1) only anatomical images (pre-RT nT2-FLAIR and T1C); (2) anatomic + diffusion images (pre-RT nT2-FLAIR, nT1C, nFA, and nADC); (3) anatomic + MRSI images (pre-RT nT2-FLAIR, nT1C, CNI, and CCrI); and (4) anatomic + diffusion + MRSI images (pre-RT nT2FLAIR, nT1C, nFA, nADC, CNI, and CCrI). All models were trained using the PCC + BCE loss, and again hyperparameter optimization was performed separately for each model.

### Hyperparameter search and model training details

Hyperparameter optimization was performed separately for each model using the Ranger optimizer, which performed and converged better than using more traditional Adam and stochastic gradient descent optimizers. All images were first normalized using min-max normalization, which resulted in better performance compared to z-score normalization. Data augmentation included random flipping and rotation per batch, adding Gaussian noise, as well as channel shuffling and channel dropping. Hyperparameter searching was performed systematically in two steps. A grid search was first employed to identify the optimal range for each hyperparameter, followed by performing 30 experiments of random search within the identified ranges. The hyperparameters subject to optimization included the number of base features, learning rate, and mini-batch size. Model selection was based on the maximization of the Tversky coefficient at $\alpha$ = 0.02 and $\beta$ = 0.98, a metric chosen for its sensitivity to both precision and recall in imbalanced datasets. This two-step process was only performed once to generate the optimal model, with resulting optimal hyperparameters identified as: learning rate = 5x10^-5^, 48 base number of features, and mini-batch size = 1. For the image input modality model comparison shown in Fig. 2, a streamlined process containing 15 iterations of random search, omitting the initial grid search was performed. The resulting hyperparameters used to train each of these models in Fig. 2 were as follows:

- Anatomic only model: learning rate = 1 × 10^–5^, 64 features, mini-batch size = 1
- Anatomic + Diffusion model: learning rate = 2× 10^-5^, 48 features, mini-batch size = 1
- Anatomic + MRSI model: learning rate = 8 × 10^–6^, 48 features, mini-batch size = 1
- Anatomic + Diffusion + MRSI model: learning rate = 8× 10^–5^, 48 features, mini-batch size = 1

### Model evaluation

Wilcoxon signed rank tests were used to statistically evaluate difference between models trained using different loss functions and modalities. Significant thresholds were selected at a *p* value of 0.05, and reported as *p* < 0.05, 0.01, 0.001, or 0.0001. To evaluate the performance of our deep learning CTV using multi-modal MRI on the test set, we compared 6 evaluation metrics (sensitivity, specificity, Dice coefficient, 95% Hausdorff distance, Tversky coefficient with $\alpha$ = 0.02 and $\beta$ = 0.98, and the newly-derived individualized PCC) between our deep learning CTV and two commonly utilized CTV definitions that were reviewed by an accredited Medical Physicists (HL) after removing critical structures such as the brain stem and thalamus:

1. RTOG CTV: The SOC CTV as recommended by the Radiation Therapy Oncology Group (RTOG), which includes the combined contrast-enhancing and T2-lesion plus a 2 cm uniform expansion^43,44^;
2. EORTC CTV: An overall more conservative CTV based on the more aggressive combination of the European Organization for Research and Treatment of Cancer (EORTC) recommendations^41^, which includes the residual contrast-enhancing tumor and resection cavity plus a 1.5 cm isotropic margin^53^ and excludes any vasogenic edema observed on the T2-FLAIR image^42^.

## Supplementary information

Supplementary information

## Data Availability

The imaging dataset used in this study cannot be shared publicly due to patient privacy and ethical restrictions. However, the results generated from this study can be made available from the corresponding author upon reasonable request.

## Abbreviations

AA: Anti-angiogenic
ADC: Apparent diffusion coefficient
AUC: Area under the receiving operating characteristic curve
ATT: Bevacizumab (Avastin)
BCE: Binary cross entropy
CEL: Contrast enhancing lesion
CCrI: choline-to-creatine index
Cho: Choline
CNI: choline-to-NAA index
Cre: Creatine
CrNI: creatine-to-NAA index
CTV: Clinical target volume
DL CTV: CTV generated using our deep learning method
DTI: Diffusion tensor imaging
DWI: Diffusion-weighted imaging
ENZA: Enzastaurin
EORTC: European Organization for Research and Treatment of Cancer
EORTC CTV: CTV generated using the EORTC recommendation, [T1C + 1.5 cm]
FA: Fractional anisotropy
GBM: Glioblastoma
Lac: Lactate
Lip: Lipid
^1^H-MRSI: Proton Magnetic Resonance Spectroscopic Imaging
NAA: N-acetylaspartate
NAV: Normal appearing voxels
NEL: Non-enhancing lesion
OS: Overall survival
PCC: Progression Coverage Coefficient
PFS: Progression-free survival
RANO: Response assessment in neuro-oncology
rCBV: relative cerebral blood volume
RF: Random Forest
ROI: Region of interest
RT: Radiation therapy
RTOG: Radiation Therapy Oncology Group
RTOG CTV: CTV generated using RTOG recommendation, [FLAIR + 2 cm]
SOC: Standard-of-care
T1C: T1 post-contrast
T2L: T2 FLAIR hyperintensity lesion

## Code availability

Code will be made available upon reasonable request according to the guidelines established by UCSF and agencies that provided funding for this study once all studies using the data have been published.
