# Supplementary material for: Novel radiotherapy target definition using AI-driven predictions of glioblastoma recurrence from metabolic and diffusion MRI
Source: NPJ Digit Med. 2025 Aug 7;8:508. doi: 10.1038/s41746-025-01861-2 (PMC12331921; doi:10.1038/s41746-025-01861-2)
Supplement: Supplementary file 1 — Supplementary information [file 41746_2025_1861_MOESM1_ESM.pdf]

Supplementary Information

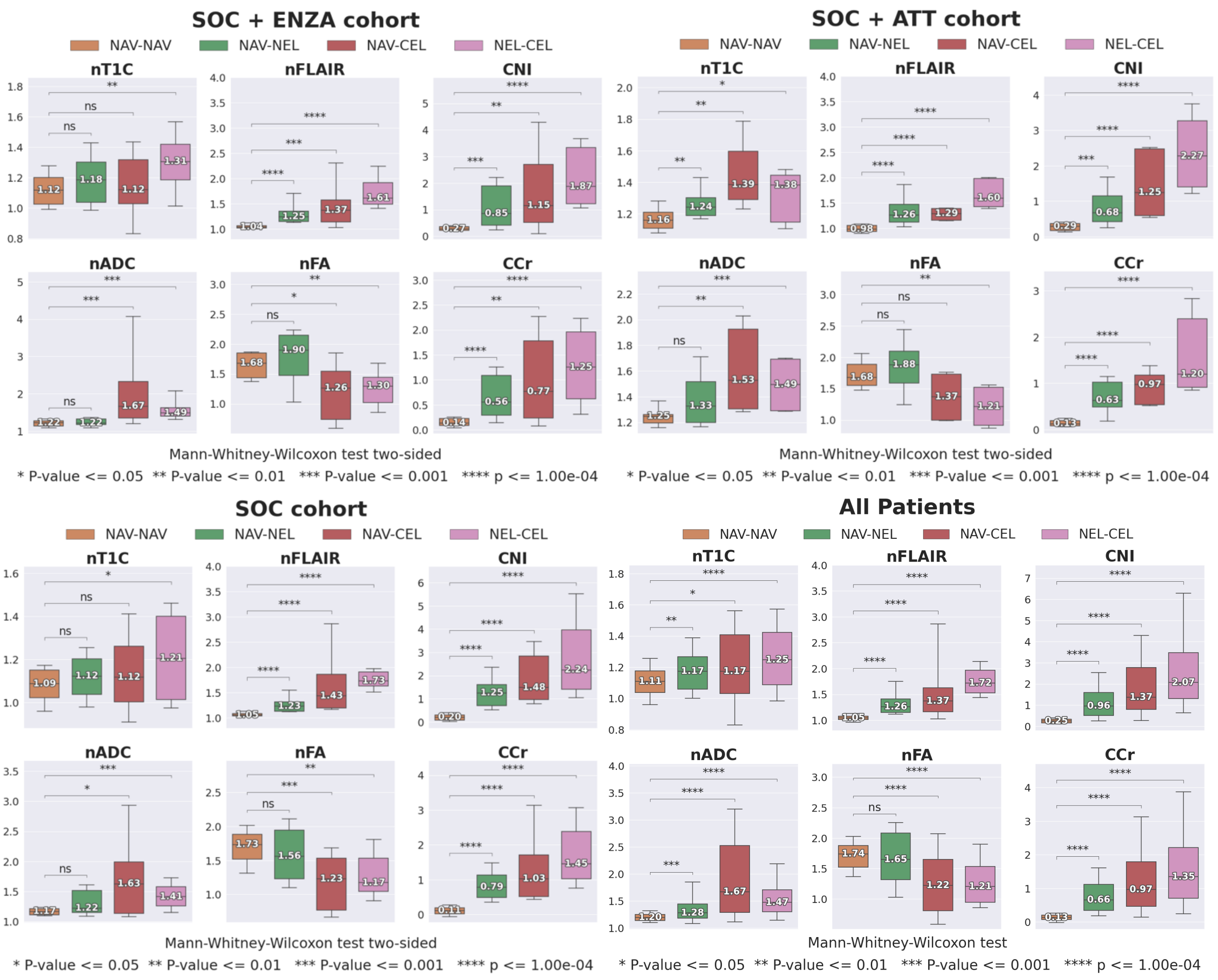

Supplementary Figure 1. Region level analyses by therapy group. Statistical comparison of MRI parameters between stable voxels (NAV→NAV) and progressed voxels (NAV→NEL, NAV→CEL, NEL→CEL) for each therapy group. (p<.05*, 0.01**, 0.001**, or 0.0001****). We found that both the metabolic and diffusion parameters were predictive of infiltrative tumor quite similarly in all 3 cohorts.

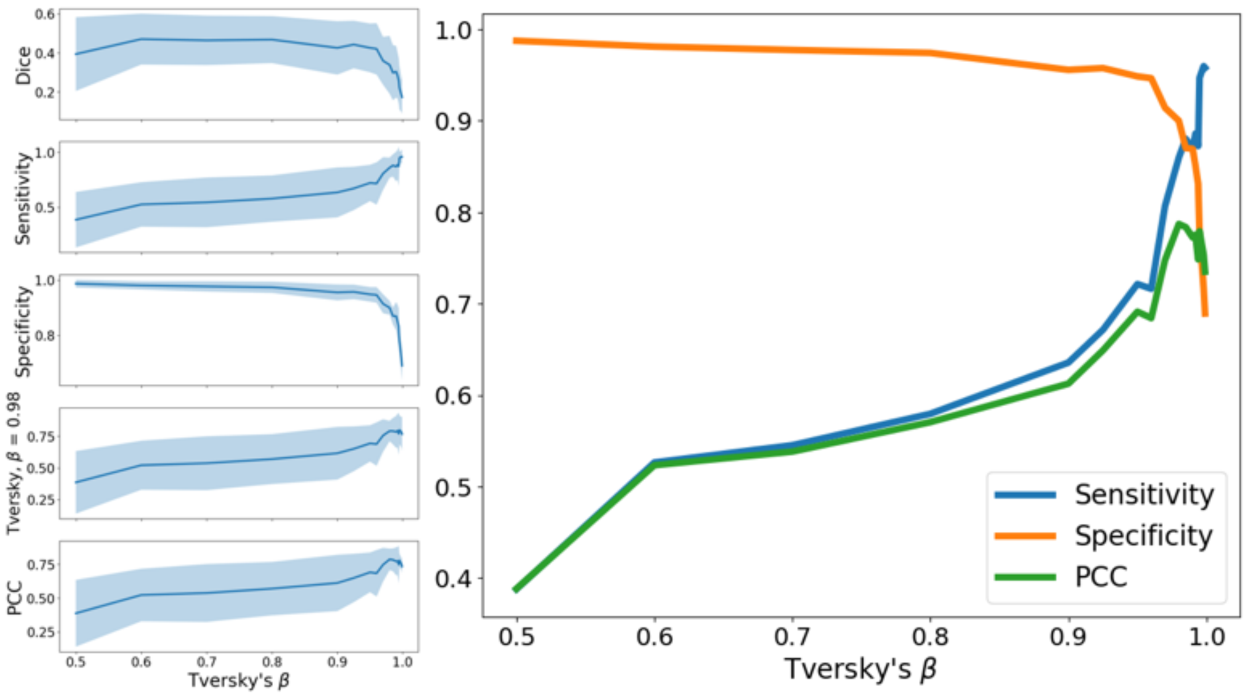

Supplementary Figure 2. Model performance vs. Tversky's β in loss function. As Tversky's β increased in the loss function, the model became more sensitive but less specific. Optimal Tversky's α and β occurred near the point where α approximately equals the average percentage of tumor voxels to the total brain voxels ($\alpha \sim 0.02$ and $\beta \sim 0.98$). Thus, we designed a loss function and evaluation metric, PCC, that used this ratio.

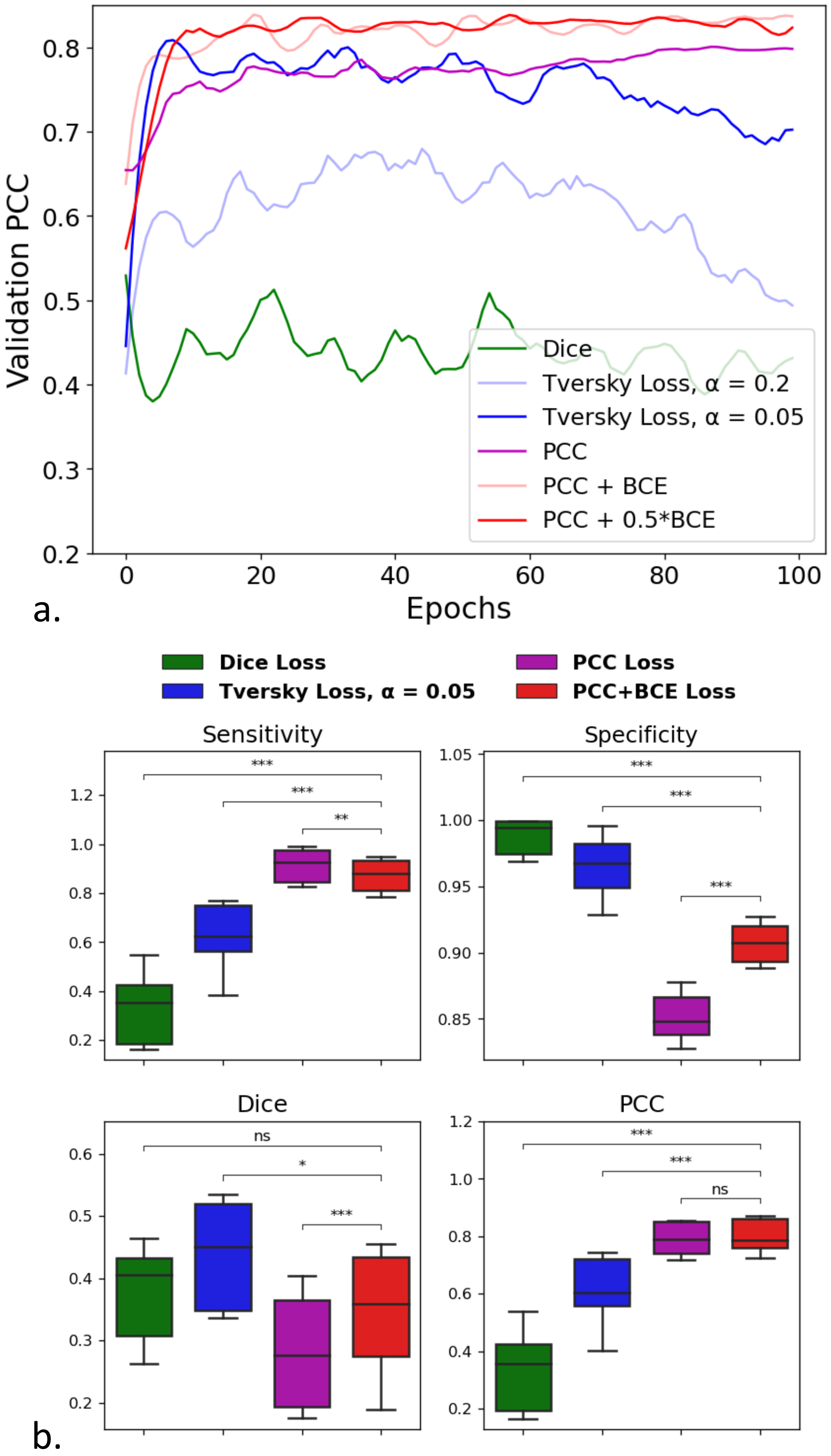

Supplementary Figure 3. Comparison of models with different loss functions. (a) The PCC score during training of all models for patients in validation set; and (b) Sensitivity, specificity, Dice, and PCC of models trained with different loss functions for patients in the test set. Significant levels from a Wilcoxon rank sum test were defined as (*, **, *** for p-values < 0.05, 0.01, and 0.001, respectively). All models were trained and optimized separately using anatomic + diffusion + MRSI as inputs. Models trained using a combination loss function with PCC + BCE achieved much better performance than any other loss functions.

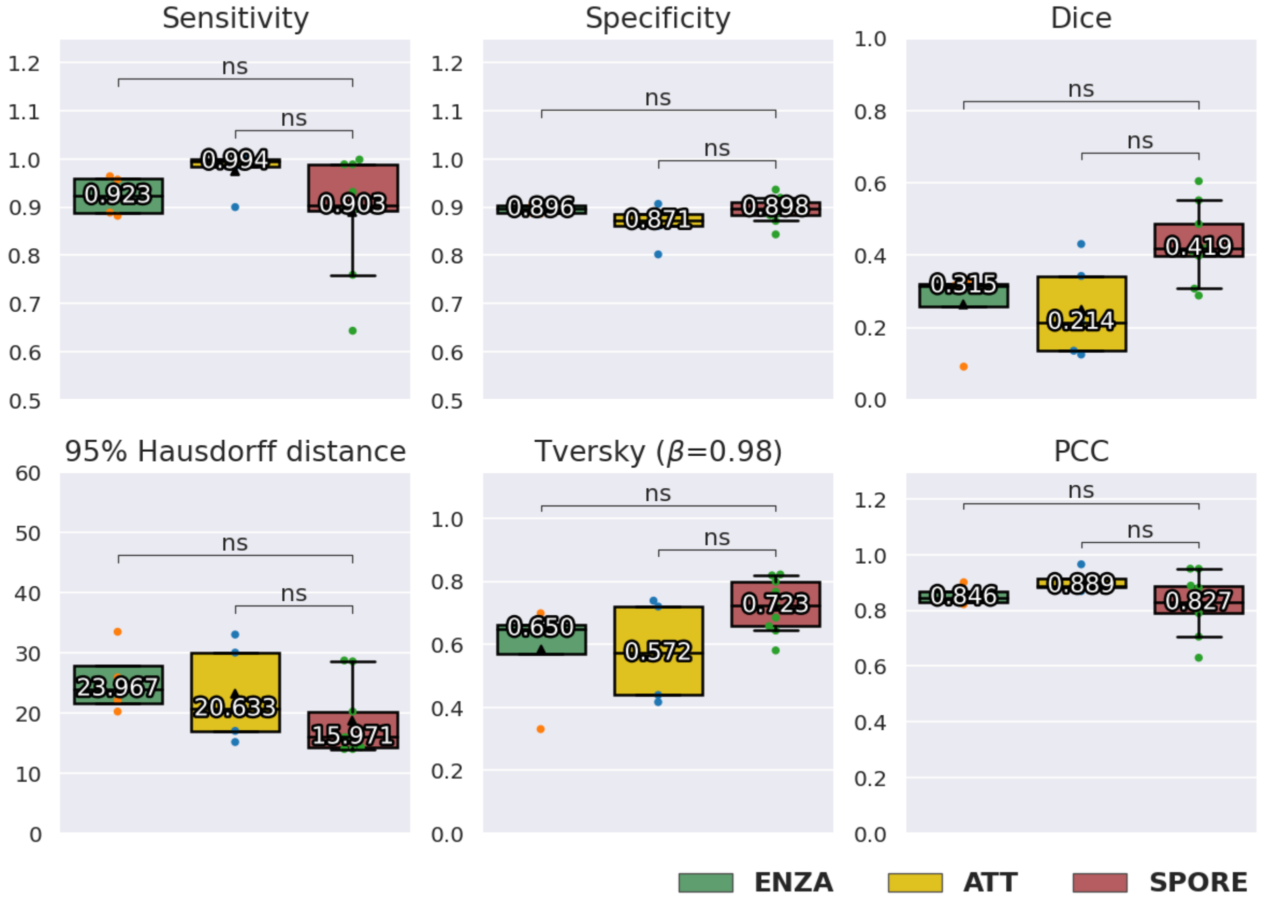

Supplementary Figure 4. Model performance by therapy group. Trends toward higher specificity, but lower sensitivity and PCC was observed in patients receiving SOC therapy compared to those who also received an anti-angiogenic agent, although the differences are not significant. Green = enzastaurin group; Yellow = bevacizumab group; red = SOC group. ns = non-significant.

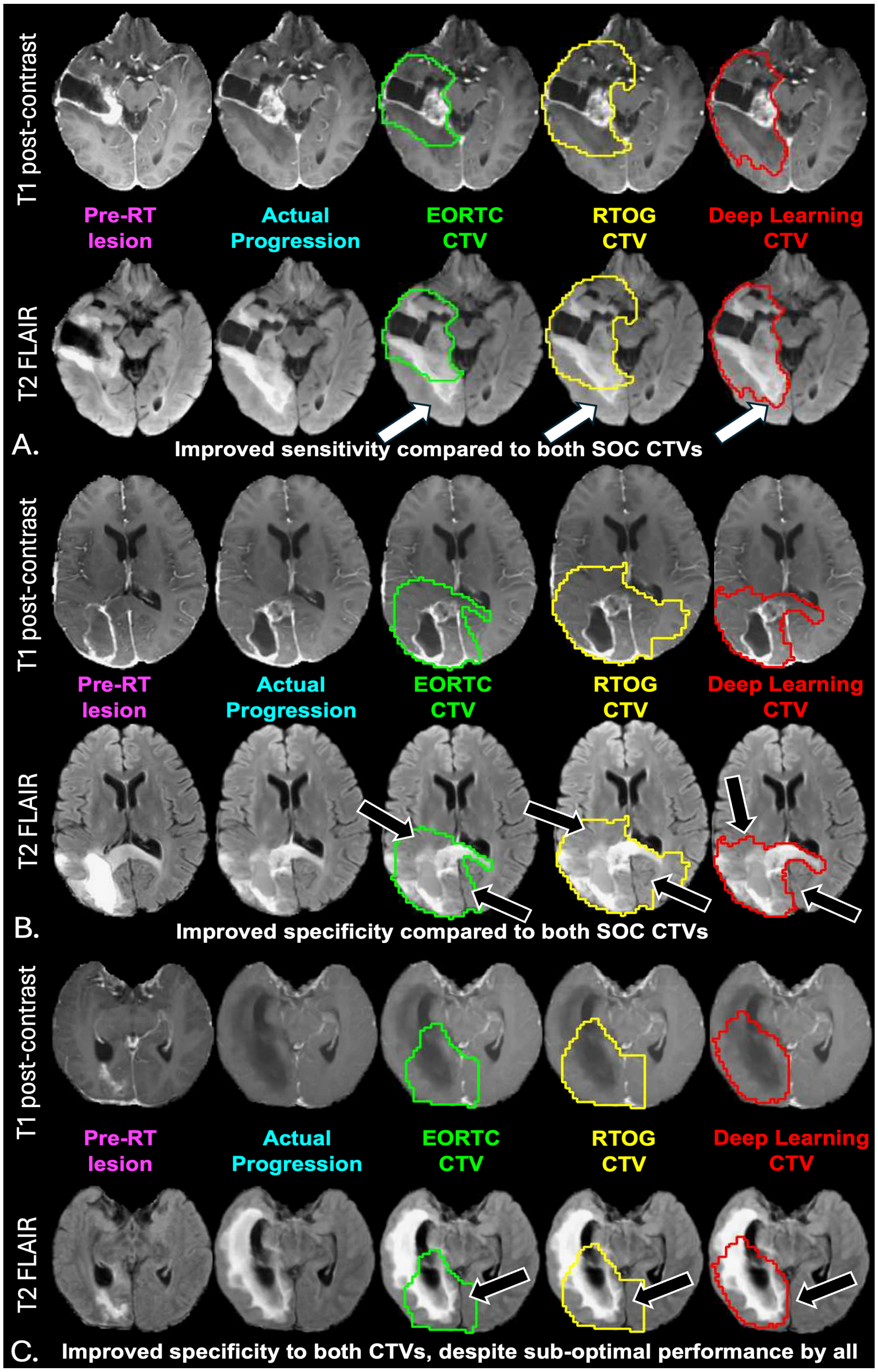

Supplementary Figure 5. CTVs for 3 additional example patients in the test set. (A) Patient #1 of Supplementary Table 2, where sensitivity was higher in our deep learning CTV compared to both other CTVs, and higher specificity was observed compared to the RTOG CTV, resulting a higher Tversky score and PCC and the CTV contour more closely following the path of tumor progression. White arrows highlight an area of the progressed lesion that was covered by our deep learning based CTV (red contour) but missed by RTOG CTV (yellow contour) (B) Patient #15 of Supplementary Table 2 follows the same trend as the patient in (A), with black arrows highlighting regions of normal brain tissue spared by our deep learning CTV compared to both EORTC (green contour) and RTOG CTVs. (C) Patient #13 of Supplementary Table 2, where our model performed the worst compared to all patients in the test set. Despite suboptimal performance, our deep learning CTV still demonstrated improved specificity, while having the same sensitivity, to the EORTC and RTOG CTVs that similarly missed covering a large portion of the progressed lesion.

Supplementary Table 1. Comparison between deep learning performance and other standard of care CTV treatment plans. Performance is recorded in mean (standard deviation), and median. The RTOG CTV treatment plan achieved high sensitivity but the lowest specificity, overtreating normal-appearing-brain. The deep learning model outperformed all other clinical CTV treatment plans in PCC, providing the best balance between high specificity and sensitivity compared to SOC CTVs. Sensitivity, specificity, Tversky, and PCC are better metrics for this task, while the conventional metrics such as Dice, Positive predicted value (PPV), and Negative predictive value (NPV) are not meaningful, since they heavily penalize false positive.

Method	Sensitivity	Specificity	Dice	95% Hausdorff distance	Tversky $\alpha = 0.02$	PCC	PPV	NPV
**Only treating pre-RT lesion**	0.35(0.15)<br>0.36	0.99(0.01)<br>1.0	0.42(0.14)<br>0.43	18.6(11.0)<br>15.9	0.36(0.15)<br>0.37	0.35(0.15)<br>0.36	0.62(0.17)<br>0.62	0.97(0.02)<br>0.98
**EORTC CTV**	0.74(0.21)<br>0.80	0.92(0.03)<br>0.93	0.37(0.12)<br>0.36	26.0(15.7)<br>19.1	0.59(0.17)<br>0.66	0.68(0.19)<br>0.75	0.29(0.07)<br>0.27	0.98(0.02)<br>0.993
**RTOG CTV**	0.95(0.08)<br>0.99	0.79(0.10)<br>0.82	0.25(0.09)<br>0.27	25.2(5.2)<br>23.0	0.59(0.12)<br>0.61	0.79(0.09)<br>0.85	0.15(0.07)<br>0.15	0.99(0.02)<br>0.999
**Deep Learning CTV**	0.92(0.11)<br>0.95	0.89(0.05)<br>0.89	0.34(0.15)<br>0.34	22.8(9.3)<br>20.2	0.63(0.17)<br>0.67	0.81(0.09)<br>0.88	0.23(0.12)<br>0.20	0.99(0.02)<br>0.999

Supplementary Table 2. Individual patient performance for different CTVs.

				EORTC CTV			***RTOG CTV***			***Deep Learning CTV***		
**Patient ID**	**PFS (days)**	**Distant Failure**	**Treatment**	**Sensitivity**	**Specificity**	**PCC**	**Sensitivity**	**Specificity**	**PCC**	**Sensitivity**	**Specificity**	**PCC**
1	98	Yes	ENZA	0.84	0.89	0.75	0.94	0.83	0.80	0.95	0.89	0.83
2	271	No	ATT	0.68	0.89	0.61	0.99	0.62	0.76	0.98	0.79	0.83
3	66	No	ENZA	0.68	0.90	0.64	0.99	0.78	0.83	0.92	0.92	0.84
4	540	Yes	ENZA	0.82	0.90	0.73	0.89	0.87	0.76	0.97	0.85	0.79
5	361	No	ATT	0.75	0.95	0.72	0.98	0.88	0.86	1.00	0.82	0.82
6	492	No	ATT	0.87	0.92	0.82	0.99	0.87	0.87	0.99	0.84	0.85
7	331	No	ATT	0.90	0.88	0.79	1.00	0.70	0.76	0.99	0.88	0.87
8	288	No	ATT	0.94	0.95	0.90	1.00	0.92	0.91	1.00	0.83	0.83
9	318	Yes	ENZA	0.85	0.93	0.77	0.90	0.92	0.81	0.96	0.85	0.82
10	67	No	SOC	0.97	0.90	0.86	1.00	0.82	0.84	1.00	0.93	0.91
11	223	No	ENZA	0.79	0.95	0.75	0.99	0.87	0.87	0.95	0.96	0.89
12	353	Yes	SOC	0.78	0.94	0.74	0.93	0.87	0.80	0.89	0.91	0.79
13	24	Yes	SOC	0.51	0.85	0.46	0.58	0.79	0.51	0.57	0.89	0.54
14	172	No	SOC	0.58	0.93	0.55	1.00	0.60	0.71	0.78	0.94	0.72
15	54	No	SOC	0.95	0.90	0.85	1.00	0.77	0.80	1.00	0.88	0.88
16	178	No	SOC	0.40	0.93	0.38	0.99	0.63	0.69	0.87	0.93	0.80
17	212	No	SOC	0.16	0.99	0.15	0.97	0.67	0.74	0.70	0.92	0.66
18	77	No	SOC	0.82	0.95	0.77	1.00	0.85	0.85	0.98	0.94	0.90
